# Conus Medullaris Syndrome following Radionuclide Cisternography

**DOI:** 10.1155/2014/201745

**Published:** 2014-06-12

**Authors:** Jay Chol Choi

**Affiliations:** Department of Neurology, Jeju National University, Aran 13 gil 15, Jeju-si, Jeju-do 690-767, Republic of Korea

## Abstract

Radionuclide cisternography is generally considered to be a safe procedure without significant neurological complications. However, in this report we present a patient who developed conus medullaris syndrome following radionuclide cisternography. A 46-year-old woman underwent lumbar puncture followed by radionuclide cisternography with the diagnosis of hydrocephalus. After the cisternography, she developed voiding difficulty with perineal sensory loss. Lumbar MRI revealed a high signal intensity lesion on T2-weighted images at the level of conus medullaris. Considering its clinical course and MRI findings, a spinal cord infarction is highly suggested as a cause of the conus medullaris lesion in this patient.

## 1. Introduction

Spinal cord lesions have been reported following spinal anesthesia, nerve root injection; the proposed mechanisms of such lesions include vascular compromise, direct needle injury, and neurotoxicity caused by the injected agents [1–6]. Radionuclide cisternography is generally considered to be a safe procedure without significant neurological complications. However, in this report we present a patient who developed the conus medullaris syndrome following radionuclide cisternography.

## 2. Case

**Figure 1:**
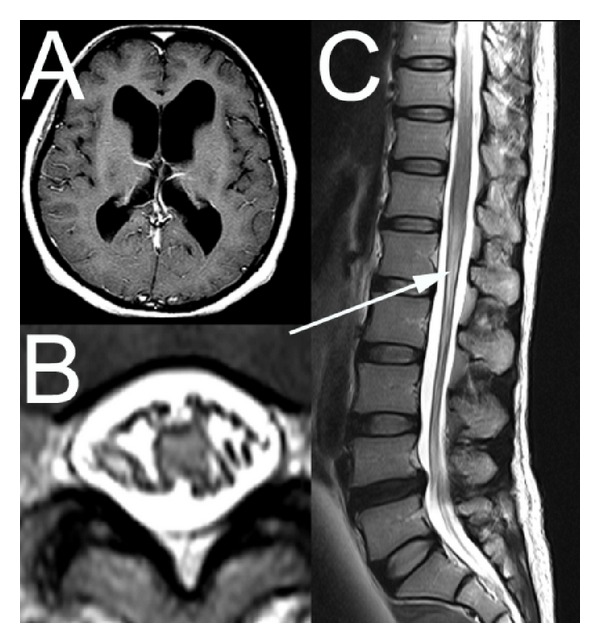
Brain MRI shows marked enlargement of all ventricles (A) and T2-weighted axial (B) and sagittal (C) MR images of the lumbar spine demonstrate increased signal intensity lesion at the level of conus medullaris (arrow). The lesion is diffuse and symmetrical and involves the whole conus medullaris on axial image.

A 46-year-old woman was admitted to the hospital because of hydrocephalus. She had suffered from a vague and subjective report of gait difficulty and dysarthria, several years before admission. A recent brain MRI, performed elsewhere, revealed enlargement of all ventricles (Figure 1(A)). On neurological examination, she was alert, and the cranial nerve functions were intact. The gait examination revealed no abnormality. The patient underwent lumbar puncture followed by radionuclide cisternography. A 22-gauge spinal needle was inserted at the L4-5 interspace. Initially she had sharp pain in the back and both legs but the pain subsided after repositioning of the needle. The color of CSF was clear, and the opening pressure was 8 cm H_2_O. Injection of 1.5 mL of ^99m^Tc-DTPA was performed uneventfully. The CSF contained no WBC. The glucose level was 53 mg per deciliter, and the total protein level was 38 mg per deciliter. Radionuclide cisternography demonstrated markedly delayed absorption of CSF with reflux into the lateral ventricles. The patient complained of voiding difficulty approximately two hours after injection. Examination revealed decreased sensation to pinprick at the perineal and perianal regions; in addition there were slightly increased deep tendon reflexes elicited from both lower extremities. No motor weakness was noted on extremities and Babinski sign was absent. A lumbar MRI demonstrated a high signal intensity lesion on T2-weighted images at the level of conus medullaris (Figures 1(B) and 1(C)). The lesion showed mild enhancement along the pial surface after gadolinium injection. Because the patient did not have a fever or headache and to prevent further neurological damage, a diagnostic lumbar puncture was not performed. Neurologic findings remained unchanged 12 months later.

## 3. Discussion

There have been several reports of aseptic meningitis following radionuclide cisternography [7, 8]. The main clinical symptoms were high fever, headache, stiff neck, and increase of cell count and of protein in CSF. Complete recovery within a few days had been the rule. Because this patient presented with sudden onset conus medullaris syndrome without fever or headache, it seems to be reasonable that her neurological deficit was not associated with aseptic meningitis or spinal arachnoiditis. Moreover, the lumbar MRI did not show clumping of nerve roots which are frequently seen in patients with arachnoiditis [9].

Spinal anesthesia is known to cause damage to the conus medullaris. Incorrect needle placement and neurotoxicity of injected agents are possible causes of such damage [1, 3, 6]. For our patient, we performed lumbar X-ray after placing a metal pin over the skin puncture mark and it revealed a metal pin at L4/5 interspace. Therefore, we excluded the possibility of spinal cord damage by direct needle injury.

A sudden clinical onset and an increased signal intensity lesion on T2-weighted images in this patient suggest a vascular event involving the spinal cord. There are an increasing number of reports on spinal vascular complications following spinal nerve root injection [2, 4, 5, 10]. The proposed mechanisms include direct vascular injury such as penetration or vasospasm induced by spinal needle, embolic distal vessel occlusion after intra-arterial injection, and mechanical compression by injected volume, or in situations with altered vascular anatomy.

Although clear CSF was withdrawn before injection of radionuclide and the injection was made inside the intradural space, the possibilities of intra-arterial injection cannot be excluded in this patient since the aspiration test alone frequently missed intravascular injection during lumbar spinal block [11]. Moreover, direct vascular damage or vasospasm induced by spinal needle might occur especially when the patient moves briefly due to sudden sharp pain in the back and both legs.

Recently, neurotoxicity of the radionuclide on conus medullaris was suggested as a possible cause of the conus medullaris syndrome following radionuclide cisternography in four Korean patients [12]. However, it is unclear why the toxic effect occurred only in the conus medullaris. The patients developed conus medullaris syndrome usually 3-4 days after the cisternography, and half of them showed mild enhancement around conus medullaris following gadolinium enhancement. Three of them improved completely within several days or months. Since the time interval between cisternography and onset of symptoms and clinical courses were different from those of the current patient, the conus medullaris syndrome following radionuclide cisternography may present with diverse spectrum.

In conclusion, this patient showed that spinal cord lesions can develop following radionuclide cisternography. Although the exact mechanism underlying such lesions is unclear, a spinal cord infarct is suggested by the clinical course and MRI findings. To minimize the risk of neurovascular complications associated with percutaneous spinal procedures, more widespread use of real-time fluoroscopy as well as contrast enhancement is encouraged [11]. Future studies will be required to provide risk factors that lead to such spinal cord lesions following a variety of spinal procedures.
